# Supplementary material for: General practitioners’ perceptions of using virtual primary care during the COVID-19 pandemic: An international cross-sectional survey study
Source: PLOS Digit Health. 2022 May 16;1(5):e0000029. doi: 10.1371/journal.pdig.0000029 (PMC9931239; doi:10.1371/journal.pdig.0000029)
Supplement: Attachment — Submitted filename: Article comments.pdf [file pdig.0000029.s002.pdf]

270 Participants mentioned organisational culture as an impediment against using virtual care.

273 Participants also reported technical

> Do you mean all participants in every country mentioned organisational culture as an impediment against using virtual care?

> Participants also reported technical issues with the digital systems used, including inadequate equipment and infrastructure, and poor systems, which participants (male, female, country)? Or overall percentage from the selected participants?

274 issues with the digital systems used, including inadequate equipment and infrastructure, and poor systems

275 interoperability.

> It is not clear which countries are related to the statement in lines 274-275. Can I conclude that all countries included in this study have same issues (inadequate equipment and infrastructure, and poor systems interoperability) ?

281 GPs identified a range of legal and regulatory weaknesses, highlighting that existing legislation is no longer

282 fit for purpose, nor meets the evolving needs resultant from the pandemic

> Which GPs. How these GPs are distributed based on your selected characteristics. What is the percentage of countries that have legal and regulatory weaknesses ? do you mean that all countries or X% have legal and regulatory weaknesses?

286 Benefits for quality of care were identified for the six dimensions of quality of care: safety, effectiveness and equity, efficiency, improved timeliness and patient-centredness.

295 Important challenges have also been noted for six domain of quality of care: patient-centredness, equity, safety, timeliness, effectiveness, and efficiency.

> It is not clear why authors use six dimensions of quality of care and six domains of quality of care? As six dimensions of quality of care and six domains of quality of care contain almost words.

> Do you mean six domain or six domains (plural)?

325 Only GPs were included in this study; future research should focus on the inclusion of other healthcare

326 professionals, and especially patients

> What are other health care professionals. I understood that “Especially patients” are one of other health care professionals or health care professionals that are patients. Can you please rewrite it to avoid this confusion? “Especially” term creates confusion. If the term “especially” will be left out, then I will create no confusion.

313 recommendations was developed based on the main findings, to support providers and healthcare

315 The results must be interpreted considering some limitations. Our findings are impacted by common

332 evaluate potential changes observed is critical to validate these findings.

414 Our findings highlight the complexity inherent to the ….,

> Authors used findings in lines 313, 315, 332 and 414 without clearly define their findings in their paper. It is not easily to point out the findings of which the authors talk about in their paper.

> Caption in Fig 2: "Conceptual map of main challenges identified" is not clear. The figure 2 is not clear.

Overall, the above questions are related to minor issues. The paper is of high quality that covers interesting topic in digital health. I salute the authors for their excellent work.
