# Supplementary material for: General practitioners’ perceptions of using virtual primary care during the COVID-19 pandemic: An international cross-sectional survey study
Source: PLOS Digit Health. 2022 May 16;1(5):e0000029. doi: 10.1371/journal.pdig.0000029 (PMC9931239; doi:10.1371/journal.pdig.0000029)
Supplement: Attachment — Submitted filename: Response to Reviewers.docx [file pdig.0000029.s003.docx]

**Response to Reviewers**

**Comment 1: Participants mentioned organisational culture as an impediment against using virtual care. Participants also reported technical issues - do you mean all participants in every country mentioned organisational culture as an impediment against using virtual care? Participants also reported technical issues with the digital systems used, including inadequate equipment and infrastructure, and poor systems, which participants (male, female, country)? Or overall percentage from the selected participants?**

Answer: Thank you for your comment. These sentiments were expressed by participants from several countries included in our study.

Concerning the quantification of the identified themes or participants who expressed certain views, while we agree would be interesting and add an additional layer of complexity to the work, that approach would not be advisable in the context of a qualitative thematic analysis, which is not powered for those uses. While in other methods (i.e., Qualitative Content Analysis), proportions or frequencies are sometimes reported, thematic analysis entails a purely qualitative account of data [1]. Qualitative samples are optimised for interpretative research and are not necessarily powered for quantitative purposes. Such quantitative analyses may lead to potentially flawed conclusions and misinterpretation of findings. As such, we decided to focus this paper on the interpretative angle.

However, we agree that a Quantitative Content Analysis could provide important insights and have highlighted this as future work needed. The edited version of the manuscript currently reads as follows: “Further qualitative content analysis could provide novel insights on their relative importance for individual countries, and specific groups of GPs.”

**Comment 2: issues with the digital systems used, including inadequate equipment and infrastructure, and poor systems interoperability. It is not clear which countries are related to the statement in lines 274-275. Can I conclude that all countries included in this study have same issues (inadequate equipment and infrastructure, and poor systems interoperability) ?**

Answer: While these challenges were widely expressed by participants in our survey, our study method did not permit us to investigate further the exact quantitative impact, or variances between participating countries. As discussed before, Quantitative Content Analysis could provide important insights and have highlighted this as future work needed. The edited version of the manuscript currently reads as follows: “Further qualitative content analysis could provide novel insights on their relative importance for individual countries, and specific groups of GPs.”

**Comment 3: GPs identified a range of legal and regulatory weaknesses, highlighting that existing legislation is no longer fit for purpose, nor meets the evolving needs resultant from the pandemic Which GPs. How these GPs are distributed based on your selected characteristics. What is the percentage of countries that have legal and regulatory weaknesses? Do you mean that all countries or X% have legal and regulatory weaknesses?**

Answer: We thank the reviewer for this comment. As described above, this manuscript describes findings that were sampled and analysed using a qualitative methodological approach. Our qualitative study design is not powered to calculate exact proportions of which GPs from which country have expressed which perspectives and may result in misinterpretations as to the strength and representativeness of the findings themselves.

Nevertheless, we do agree on the importance of future quantitative studies that adequately evaluate the views expressed and quantify their prevalence or magnitude amongst healthcare providers and in what international settings. This point is emphasised in the manuscript as follows:

*“While our results do not allow to draw specific conclusion on this matter, future research should aim to clarify geographic variations, within and between countries, in what concerns the availability and use or virtual primary care. […]*

*It is also important to note that this study evaluates qualitatively GPs perceptions on the impact of remote care; future research using quantitative approaches to objectively evaluate potential changes observed is critical to validate these findings.”*

**Comment 4: Benefits for quality of care were identified for the six dimensions of quality of care: safety, effectiveness and equity, efficiency, improved timeliness and patient-centredness. Important challenges have also been noted for six domain of quality of care: patient-centredness, equity, safety, timeliness, effectiveness, and efficiency. It is not clear why authors use six dimensions of quality of care and six domains of quality of care? As six dimensions of quality of care and six domains of quality of care contain almost words. Do you mean six domain or six domains (plural)?**

Answer: Thanks for noting this and we apologise for the inconsistency. We have now reviewed the manuscript and adopted the use of the term domains consistently across the document, abiding by the terminology defined by the Institute of Medicine [2,3]. Also thank you for bringing this typographic error (plural) to our attention.

**Comment 5: Only GPs were included in this study; future research should focus on the inclusion of other healthcare professionals, and especially patients. What are other health care professionals. I understood that “Especially patients” are one of other health care professionals or health care professionals that are patients. Can you please rewrite it to avoid this confusion? “Especially” term creates confusion. If the term “especially” will be left out, then I will create no confusion.**

Answer: Thank you for highlighting this issue. The sentence has now been rewritten and adjusted to aid in clarity as suggested. The edited version of the manuscript now reads as follows:

*“Only GPs were included in this study; future research should focus on the inclusion of other healthcare professionals and patients.”*

**Comment 6: “recommendations was developed based on the main findings, to support providers and healthcare, The results must be interpreted considering some limitations. Our findings are impacted by common evaluate potential changes observed is critical to validate these findings. Our findings highlight the complexity inherent to the ….,: Authors used findings in lines 313, 315, 332 and 414 without clearly define their findings in their paper. It is not easily to point out the findings of which the authors talk about in their paper.**

Answer: Thank you for your comment concerning this issue. The principal findings section has now been rewritten in hopes of bringing greater clarity for the reader. The revised text now reads as follows:

*“Benefits were identified in accordance with the six domains of quality of care. These included a reduction in exposure risks for COVID-19 transmission, ensuring access and continuity of care to those who need it, including those who had previously limited access to face-to-face consultations, enabling remote triage, in addition to improved patient convenience, communication, and empowerment. Benefits for healthcare providers included a greater work flexibility and more control over their schedules. Benefits for health systems included hastening the digital transformation through increasing awareness, trust, adoption, skills, and technical capacity, as well as driving changes in legal and regulatory frameworks.*

*Likewise, significant challenges have also been highlighted across the six domains of quality of care. These included patients’ preference for face-to-face care, the potential negative impact on communication, and the lack of equipment, internet access and digital skills of some patient groups. In addition, clinical uncertainty and potentially inappropriate decision making resulting in delays in diagnosis and treatment, unsuitability for certain consultations, as well as overuse and misuse of healthcare resources, were also mentioned. Challenges specific to healthcare providers included the lack of guidance and support, higher workload, and remuneration issues. From the health systems’ perspective, the long-established organisational culture, technological difficulties, implementation and financial issues, and inadequate accompanying supportive policies and regulatory legislation, were also challenges described by participants.”*

**Comment 7: Caption in Fig 2: "Conceptual map of main challenges identified" is not clear. The figure 2 is not clear.**

Answer: Thank you for your comment in highlighting this issue. The caption has now been updated to include a more detailed description of the figure. The caption now reads as follows:

*“Fig 2: Conceptual map of the main challenges surrounding the use of virtual care tools for primary care during the initial months of COVID-19 identified.”*

A higher resolution version of the figure will also be uploaded in this revision alongside the textual changes.

**Comment 8: Overall, the above questions are related to minor issues. The paper is of high quality that covers interesting topic in digital health. I salute the authors for their excellent work**

Answer: Thank you for taking the time to review our manuscript and for your encouraging comments on our work.

**References**

1	Vaismoradi M, Snelgrove S. Theme in Qualitative Content Analysis and Thematic Analysis. *Forum Qual Sozialforsch / Forum Qual Soc Res* 2019;**20**. doi:10.17169/FQS-20.3.3376

2	Institute of Medicine (US) Committee on Quality of Health Care in America. Six Domains of Health Care Quality | Agency for Healthcare Research and Quality. https://www.ahrq.gov/talkingquality/measures/six-domains.html (accessed 3 Feb 2022).

3	Institute of Medicine (US) Committee on Quality of Health Care in America. Crossing the Quality Chasm: A New Health System for the 21st Century. Published Online First: 19 July 2001. doi:10.17226/10027
